# Partial Breast Irradiation for Early-Stage Breast Cancer: Advances, Challenges, and Future Directions—A Narrative Review

**DOI:** 10.3390/tomography11060059

**Published:** 2025-05-22

**Authors:** Ayyaz Qadir, Nabita Singh, Anelyn Chui, Michael Chao, Sergio Uribe, Farshad Foroudi

**Affiliations:** 1 Department of Medical Imaging and Radiation Sciences, School of Primary and Allied Health Care, Monash University, Melbourne, VIC 3800, Australia; 2 Sir Peter MacCallum Department of Oncology, The University of Melbourne, Parkville, VIC 3010, Australia; 3 Department of Radiation Oncology, Olivia Newton-John Cancer Wellness and Research Centre, Austin Health, Heidelberg, VIC 3084, Australia; 4 Genesis Care Victoria, Ringwood Private Hospital, 36 Mt Dandenong Rd., Ringwood East, VIC 3135, Australia

**Keywords:** breast cancer, review, radiotherapy, magnetic resonance imaging-guided radiotherapy

## Abstract

Advances in breast cancer treatment have shifted the focus from maximizing local control to balancing oncologic efficacy with treatment de-escalation and toxicity reduction. Whole-breast irradiation (WBI) following breast-conserving surgery remains the standard of care, but with up to 90% of recurrences occurring near the tumor bed, partial breast irradiation (PBI) has emerged as a viable alternative. Large randomized controlled trials (such as IMPORT LOW, Florence, and GEC-ESTRO) have demonstrated comparable ipsilateral breast tumor recurrence (IBTR) rates between PBI and WBI, reinforcing its oncologic safety in well-selected patients. However, challenges remain in optimizing fractionation schedules, refining patient selection, and minimizing late toxicity. Recent innovations, including MRI-guided radiotherapy (MRgRT) and neoadjuvant PBI, offer improved tumor targeting, real-time plan adaptation, and enhanced normal tissue sparing. These advancements hold promise for further reducing radiation-related morbidity and improving cosmetic outcomes. As PBI progresses, integrating novel imaging modalities and hypofractionated regimens will be crucial to refining protocols. This review synthesizes the latest evidence on PBI techniques, clinical outcomes, and emerging technologies to guide future research and clinical decision-making in precision breast radiotherapy.

## 1. Background

Breast cancer is the most common malignancy among women worldwide, with approximately 2.3 million new cases diagnosed annually [1]. Advances in early detection and systemic therapies have significantly improved survival, with global 5-year survival rates increasing from 67.9% to 78.2% over the past decade [2,3,4]. As survival improves, breast cancer management has expanded beyond disease control to prioritizing both short-term and long-term quality of life for survivors [5].

Breast-conserving therapy (BCT), which combines breast-conserving surgery (BCS) with whole-breast irradiation (WBI), has become the standard of care for early-stage breast cancer following landmark trials demonstrating its oncologic equivalence to mastectomy [6,7]. Beyond maintaining survival outcomes, BCT preserves the breast, improving psychosocial well-being [8,9,10]. However, traditional WBI regimens—typically delivering 50 Gy over 5–6 weeks—pose logistical and accessibility challenges, particularly for older patients, those in rural areas, and individuals with limited healthcare access [5,11]. Consequently, up to 30% of women undergoing lumpectomy do not receive the recommended adjuvant radiotherapy, highlighting the need for more accessible, patient-centered treatment approaches [11]. To address these challenges, hypofractionated WBI has been widely adopted, delivering higher doses per fraction over a shorter duration while maintaining efficacy and reducing toxicity [12]. The UK START trials [13,14] provided much of the pivotal evidence supporting this approach, demonstrating that a 3-week regimen of 40 Gy in 15 fractions was equivalent to the conventional 50 Gy in 25 fractions in terms of local control and toxicity, leading to its widespread adoption [15]. Building on this, ultra-hypofractionation has emerged as a further refinement, further reducing treatment burden while maintaining oncologic efficacy. The FAST-Forward trial demonstrated that in low-risk breast cancer subtypes, WBI delivered in just 5 fractions over one week (26 Gy in 5 fractions) was non-inferior to the standard 40 Gy in 15 fractions, with comparable local recurrence rates at five years and similar late toxicity [16]. However, long-term follow-up data are still awaited, particularly to assess the durability of disease control and late effects. Furthermore, additional studies are needed to determine whether a simultaneous integrated boost can be safely and effectively incorporated into the five-fraction schedule. Despite these advancements, minimizing radiation exposure to adjacent organs remains a challenge. In left-sided breast cancer, WBI, especially with nodal coverage, is associated with an increased risk of ischemic heart disease and pulmonary fibrosis due to incidental dose exposure to the heart and lungs [17,18]. To mitigate these risks, partial breast irradiation (PBI) has emerged as a promising alternative, targeting radiation specifically to the lumpectomy cavity—the highest risk zone for recurrence. Studies show that approximately 90% of local recurrences in early-stage breast cancer occur within or near the lumpectomy site (within 1–2 cm), whereas recurrences elsewhere in the ipsilateral breast are rare [19,20]. By focusing treatment on the primary site of recurrence risk, PBI reduces radiation exposure to healthy breast tissue and nearby organs, including the lungs, heart, and ribs [6,7,21]. This targeted approach allows for the delivery of higher radiation doses to the high-risk zone while sparing surrounding normal tissues.

PBI can shorten treatment duration, improving patient adherence and enhancing treatment convenience [22]. Multiple PBI delivery techniques have been developed, including brachytherapy, proton beam therapy, intraoperative radiotherapy, and external beam radiation therapy, with treatment durations ranging from one to three weeks. However, key uncertainties remain regarding the optimal radiotherapy technique and fractionation schedule to balance efficacy and toxicity. Furthermore, emerging advancements in radiotherapy, such as MRI-guided radiotherapy (MRgRT) and neoadjuvant (preoperative) PBI, offer promising opportunities to additionally improve treatment efficacy.

The aim of this review is to evaluate the development of PBI by examining its evolution, the techniques that have led to its adoption in select breast cancer populations, and its associated benefits and risks. Additionally, it seeks to address critical knowledge gaps and provide insights to guide future clinical practice and research.

## 2. Delivery Methods for PBI

### 2.1. PBI Techniques

PBI can be delivered through various techniques, including external beam radiotherapy (EBRT) using linear accelerators (LINACs); proton beam therapy, which uses positively charged protons accelerated by a cyclotron or synchrotron to deposit a dose with high precision; or brachytherapy, which involves implanting radiation sources within and directly around the lumpectomy cavity via catheters [11,23,24,25]. The choice of technique depends on multiple factors, such as patient anatomy, physician expertise, resource availability, and patient preferences. Below, we provide an overview of the commonly used PBI modalities, outlining their clinical applications, advantages, and limitations.

### 2.2. Brachytherapy

Interstitial multicatheter brachytherapy (IMBT) is one of the earliest PBI techniques, with large randomized controlled trials (RCTs) evaluating its use compared to WBI [24,26,27]. It delivers radiation directly to the tumor bed via multiple catheters implanted either perioperatively or postoperatively following BCS. High-dose-rate brachytherapy (HDR) is then administered through afterloading radioactive sources, with the catheter being removed upon treatment completion [28]. A key advantage of IMBT is its ability to deliver precise radiation doses with a steep dose fall-off, minimizing exposure to surrounding healthy tissues. This targeted delivery enables hypofractionation, allowing treatment to be completed in an accelerated schedule, typically involving 30 to 34 Gy delivered over 7 to 10 days [26,27,28,29,30]. However, the technical complexity of IMBT lies in the need for multiple catheter placements and intricate dosimetric calculations, which require a high degree of expertise [28,29]. Additionally, the steep dose gradients inherent to IMBT can create hotspots within the treatment volume, increasing the risk of complications such as fat necrosis and subcutaneous toxicity compared to EBRT [23,28,29,31,32]. Intracavitary brachytherapy offers a less technically demanding alternative involving the use of the MammoSite balloon brachytherapy applicator (Proxima Therapeutics, Alpharetta, GA, USA) [28]. This method employs a single-lumen balloon catheter inserted into the tumor bed during BCS, with radiation delivered postoperatively, typically in 10 fractions over 5 to 7 days, administered twice daily [23,28,29]. While intracavitary brachytherapy does not require the precise placement of multiple catheters like IMBT, it shares many of the same challenges, including catheter misplacement and procedural complications [23]. However, unlike IMBT, intracavitary brachytherapy has very little phase III data supporting its clinical use, limiting widespread adoption. Despite differences in complexity, both IMBT and intracavitary brachytherapy share common limitations, most notably their invasive nature. In both techniques, perioperative pain, infection, and catheter misplacement are potential risks. Although rare, these complications can lead to geographic miss of the target volume, increasing the risk of local recurrence [23,24]. Both approaches also require significant expertise and specialized resources as optimal outcomes depend on precise catheter placement and treatment planning, resulting in a steep learning curve for physicians [23,28,29]. While both techniques carry risks, IMBT is generally considered more complex due to the need for multiple catheters and intricate dosimetric calculations, whereas intracavitary brachytherapy involves a simpler, single-catheter approach. Acute side effects, such as localized inflammation and irritation, are common in both techniques due to the high radiation dose but are typically transient [11,23,26,27,28,29,33].

#### 2.2.1. Intraoperative Radiation Therapy (IORT)

IORT integrates radiotherapy into the surgical workflow by delivering a single dose of radiation during surgery [31,34,35,36,37]. It can be administered using low-energy X-rays (e.g., Intrabeam) or electrons, each with distinct advantages. Low-energy X-ray IORT is feasible in standard operating rooms, while electron IORT requires specialized equipment including a shielded linear accelerator setup [22,23,31,37].

Both approaches aim to minimize radiation exposure to surrounding normal tissues, but they also present challenges. IORT has been associated with a higher risk of local recurrence compared to WBI, particularly in long-term follow-up and multicenter RCT subgroups. Additionally, while skin toxicity is lower with IORT, there is an increased risk of fat necrosis, edema, and scar calcification, likely due to high-dose radiation exposure to nearby non-target tissues in a single fraction [35,36,38,39,40]. Currently, IORT alone is not recommended as a definitive treatment for early-stage breast cancer due to the lack of RCT (level 1) evidence supporting its noninferiority to WBI [22,41].

#### 2.2.2. Proton Beam Therapy

Proton beam therapy is an advanced external beam technique for delivering accelerated PBI. It exploits the physical properties of the Bragg peak, enabling high-dose radiation delivery at a specific depth with rapid dose fall-off beyond the target, thereby minimizing exposure to surrounding normal tissues. This precision is particularly advantageous in patients with unfavorable anatomy or when critical organs lie close to the treatment volume [42,43]. While passively scattered proton therapy was initially used with carefully selected beam angles to improve dose conformality and reduce entrance and exit doses [25], newer techniques such as pencil beam scanning offer further improvements in dose distribution and skin sparing. Early clinical experiences with proton accelerated PBI reported mixed results, with some studies noting an increased rate of acute and late skin toxicity compared to external beam radiotherapy (EBRT) [43], while others demonstrated more favorable toxicity profiles [42,44,45]. Notably, a phase I/II trial at the Abramson Cancer Center evaluated hypofractionated proton accelerated PBI (40 Gy in 10 fractions) in 32 early-stage breast cancer patients. At a median follow-up of five years, the study reported 97.6% local control, no grade ≥ 3 acute or late toxicities, and 85% good to excellent physician-rated cosmesis, with similarly positive patient-reported outcomes [46]. More recent prospective studies using pencil beam scanning, including those from the Proton Collaborative Group and Mayo Clinic, have shown promising early results and improved toxicity profiles—particularly with hypofractionated regimens such as three-fraction schedules [47]. However, these findings remain preliminary and require validation in larger phase II trials. Despite its potential advantages, the modest dosimetric benefit of proton accelerated PBI, limited accessibility, the absence of long-term outcome data, and the lack of randomized trials currently constrain its adoption in routine clinical practice compared to EBRT [48].

#### 2.2.3. External Beam Radiotherapy (EBRT)

EBRT is the most extensively studied PBI technique, supported by numerous RCTs [30,49,50,51,52,53,54,55]. Its widespread availability and familiarity among clinical oncologists make it the most accessible and non-invasive PBI option [11]. Compared to other PBI techniques, EBRT provides a more homogeneous dose distribution, reducing localized hotspots that may contribute to fat necrosis and subcutaneous toxicity [11,22,23]. Due to its broad applicability, EBRT has become a preferred PBI modality and remains the focus of several long-term RCTs comparing PBI with WBI [49,50,51,52,55].

## 3. Clinical Evidence—Results from RCTs

Numerous RCTs have compared PBI and WBI across various techniques, patient populations, and follow-up durations. Collectively, these studies have shaped the current understanding of PBI’s role in early-stage breast cancer management. A summary of key trial results is provided in Table 1, with a brief review below.

### 3.1. Oncological Outcomes and Recurrence Rates

Large, well-designed RCTs evaluating PBI versus WBI using EBRT [49,50,51,52,55] and brachytherapy [26,27,56] consistently demonstrate comparable ipsilateral breast tumor recurrence (IBTR) rates. For instance, the IMPORT LOW [52] and Barcelona trials [49] (EBRT) reported no significant differences in 5-year IBTR rates (0.5% and 0% for PBI vs. 1.1% and 0% for WBI, respectively), while the Florence trial [50,55] showed similar findings at both 5 and 10 years (1.5% and 1.5% for PBI vs. 2.5% and 3.7% for WBI, respectively). Likewise, brachytherapy-based trials, such as Groupe Européen de Curiethérapie—European Society for Radiotherapy and Oncology (GEC-ESTRO) [26] and Budapest [27], demonstrated comparable 10-year IBTR rates (3.51% and 5.9% for PBI vs. 1.58% and 5.1% for WBI, respectively), reinforcing PBI as a safe and effective alternative to WBI. In contrast, IORT-based trials, such as TARGIT-A [35,38] and ELIOT [36], reported significantly higher IBTR rates, particularly in patients under 50 years old or those with high-risk tumor features. The ELIOT trial [36] found a tenfold higher IBTR rate for IORT (4.4%) compared to WBI (0.4%) (*p* < 0.0001), while TARGIT-A [35] observed a nearly threefold increase (3.3% vs. 1.3%). These differences likely arose from broader patient inclusion criteria, including a younger age, high-grade histology, and lymphovascular invasion, highlighting the importance of careful patient selection when considering IORT-based PBI. Despite IBTR variations, overall survival (OS) remains consistently high across all trials [26,27,30,35,36,38,49,50,51,52,53,54,55,56], confirming no survival disadvantage for appropriately selected PBI patients.

### 3.2. Cosmesis and Toxicity Profiles

Cosmetic outcomes following PBI are generally favorable or equivalent to WBI, though significant variability exists across studies [50,51,52,55]. Trials using once-daily hypofractionated EBRT regimens—such as Florence [50] and IMPORT LOW [52]—consistently report good to excellent cosmesis, along with lower late toxicity compared to WBI. Similarly, the Budapest trial [27] (interstitial brachytherapy) demonstrated a cosmetic benefit, with 81% of PBI patients achieving good to excellent cosmesis compared to 63% in the WBI arm, reinforcing the potential advantages of brachytherapy-based PBI. In contrast, a subgroup analysis of the TARGIT-A trial reported significantly higher rates of fat necrosis (56% vs. 24%) and scar calcification (63% vs. 19%) with IORT compared to WBI, highlighting potential trade-offs in toxicity and overall breast cosmesis [37,39,40]. Similarly, twice-daily fractionation schedules using EBRT—such as those in the RAPID [53] and IRMA [54] trials—have been associated with worse cosmesis and higher toxicity rates, primarily due to increased fibrosis and breast induration at 3 and 5 years. The RAPID trial [53] reported grade ≥ 2 late toxicity in 32% of PBI patients vs. 13% in the WBI group, with grade 3 toxicity occurring in 4.5% vs. 1.0% of patients, respectively. Similarly, the IRMA trial [54] observed increased late toxicity and inferior cosmetic outcomes, reinforcing concerns about twice-daily fractionation.

### 3.3. Quality of Life Outcomes

Not all included RCTs reported patient quality of life (QoL) outcomes; however, three studies did [52,57,58], with all concluding that shorter radiotherapy schedules improved Health-Related Quality of Life (HRQoL) in the PBI arm. For example, the Florence trial found significant improvements in several functional and symptom scores using the European Organisation for Research and Treatment of Cancer (EORTC) Core Quality of Life (QLQ-C30) and EORTC QLQ-BR23 questionnaires both at treatment completion and two years post-irradiation. Similarly, the IMPORT-LOW trial reported better QoL outcomes using the QLQ-BR23 questionnaire. The GEC-ESTRO trial demonstrated that brachytherapy-based PBI maintained QoL outcomes comparable to WBI.

These findings suggest that PBI, particularly when delivered via once-daily EBRT or brachytherapy-based approaches, may be a preferable alternative to WBI for eligible patients.

### 3.4. Dose and Fractionation Schedules in PBI

Current recommendations for EBRT-based PBI are primarily derived from the Florence [50,55] and IMPORT LOW [52] trials, which support the use of 30 Gy in 5 fractions (daily or every other day) or 40 Gy in 15 daily fractions. Based on the findings from the RAPID [53] and IRMA [54] trials, the American Society for Radiation Oncology (ASTRO) and the Advisory Committee in Radiation Oncology Practice (ACROP) recommend against twice-daily EBRT schedules as they are associated with increased toxicity and suboptimal cosmetic outcomes [22,59]. Although no global consensus has been established, emerging data suggest that lower-dose ultra-hypofractionated PBI regimens, such as 27.5 Gy delivered in five daily fractions, may improve cosmetic outcomes, with promising 5-year results [60]. However, long-term follow-up is needed to confirm these findings. Similarly, both the UK National Institute for Health and Care Excellence (NICE) and ESTRO-ACROP strongly endorse the use of 26 Gy in five daily fractions for PBI, following the FAST-FORWARD trial’s demonstration of non-inferiority to conventional fractionation [61,62]. Despite these endorsements, long-term outcome data are still required to fully establish the safety and efficacy of these ultra-hypofractionated low-dose PBI regimens.

For brachytherapy-based PBI, dose regimens typically range from 30 to 34 Gy in 7–10 twice-daily fractions [26,27,28,29,30]. To enhance patient adherence and reduce the workload of brachytherapy units, ongoing research is focused on developing shorter, accelerated PBI schedules without compromising oncologic safety. For example, a phase I/II trial evaluating 28 Gy in four twice-daily fractions demonstrated high local control and low late toxicity rates [63]. Similarly, the TRIUMPH-T trial assessed a three-fraction brachytherapy-based PBI regimen (total dose: 22.5 Gy) and reported low acute and late toxicity rates along with good to excellent cosmetic outcomes [64]. However, long-term data remain necessary to confirm these findings.

### 3.5. Selection of Candidates for PBI

Careful patient selection is crucial to ensuring the oncologic safety and efficacy of PBI. While randomized trials have demonstrated comparable IBTR rates between PBI and WBI in well-selected patients [26,27,30,49,50,51,52,53,55,56], its role remains uncertain in subgroups with limited representation in clinical trials. Guidelines from the American Brachytherapy Society (ABS) [41], ASTRO [22], and GEC-ESTRO [65] are summarized in Table 2. The following sections provide a detailed discussion of key factors influencing PBI eligibility, including tumor size, histologic grade, molecular subtype, and patient age.

### 3.6. Tumor Size

As tumor size is a key determinant of recurrence risk, most patients enrolled in PBI trials had tumors less than or equal to 2 cm [26,27,30,50,52,53,55,56,66]. The IMPORT LOW [52] and Florence trials [50,55] found no significant difference in IBTR rates between PBI and WBI for tumors ≤ 2 cm, reinforcing PBI’s safety in this subgroup. Fewer cases involved tumors between 2.1 and 3 cm, limiting the robustness of data in this subgroup. The NSABP-B39/RTOG 0413 [30] and RAPID [53] trials suggested a slight but not statistically significant increase in IBTR for tumors > 1.5 cm [22]. A post hoc analysis of NSABP-B39/RTOG 0413 [30] found comparable IBTR rates among patients with tumors ≤ 1 cm and those measuring 2.1–3 cm, whereas tumors between 1 and 2 cm had slightly better IBTR outcomes with WBI than PBI. Based on these findings, PBI is strongly recommended for tumors ≤ 2 cm, while tumors measuring 2.1 to 3 cm may be considered with caution. In contrast, PBI is not recommended for tumors > 3 cm given the limited evidence supporting its oncologic safety in this subgroup [22].

### 3.7. Histologic Grade

Histologic grade is a key predictor of recurrence risk as high-grade tumors exhibit increased proliferation and greater metastatic potential. Most PBI trials predominantly included grade 1 and 2 unifocal lesions without lymphovascular invasion [26,30,52,53,56], but there are limited data available for grade 3 tumors. A subset analysis of the RAPID trial [53] found no significant difference in IBTR rates between grade 3 tumors treated with PBI versus WBI after 8 years, though the small sample size limits the strength of this conclusion. Similarly, NSABP-B39/RTOG 0413 [30] included over 25% grade 3 tumors but did not perform a specific subset analysis for this group [22]. In contrast, the ELIOT trial [36] found that grade 3 tumors had a significantly higher IBTR rate after PBI, with recurrence reaching 16.8% at 15 years (*p* = 0.0019). This increased risk was likely influenced by the trial’s inclusion of a higher proportion of grade 3 tumors (~20%), underscoring a potential limitation of PBI in this subgroup. Given these findings, PBI remains a well-supported option for grade 1–2 tumors with negative surgical margins and no lymph node involvement. However, for grade 3 tumors, the increased IBTR risk observed in the ELIOT trial [36], along with limited data from other studies, suggests that PBI should be used with caution [22].

### 3.8. Breast Cancer Subtypes

Breast cancer subtypes significantly influence recurrence risk and should be carefully considered when selecting patients for PBI. Patients with ER-positive, HER2-negative tumors were well represented in PBI trials and consistently demonstrated excellent oncologic outcomes, making them the most suitable candidates [26,27,30,50,52,53,56]. In contrast, individuals with ER-negative tumors comprised a smaller proportion of enrolled patients, with NSABP-B39 comprising approximately 20% [30]. Although no clear increase in IBTR rates was observed in ER-negative tumors treated with PBI, the limited sample size prevents strong conclusions. Even less data exist for HER2-positive tumors, with fewer than one hundred patients reported across all trials [50,52,53], making it difficult to determine whether WBI provides superior local control [22]. Patients with germline BRCA1/2 mutations were specifically excluded from all PBI trials, leaving a lack of data in this disproportionately younger patient population. Given the absence of robust clinical evidence, PBI is not recommended for BRCA1/2 mutation carriers [22]. In addition to molecular subtypes, histological subtypes such as invasive ductal carcinoma (IDC), invasive lobular carcinoma (ILC), and phyllodes tumors also play a crucial role in radiotherapy selection but remain underexplored in the PBI literature. IDC is the predominant histology and has formed the basis for inclusion in most PBI trials. Conversely, ILC was specifically excluded, and where included, it represented fewer than one thousand patients [52,53,66]. ILC’s diffuse growth pattern, higher likelihood of multifocality and multicentricity, and challenges in accurately delineating the tumor bed raise concerns regarding the adequacy of limited field irradiation [22,67,68]. Similarly, phyllodes tumors, although rare, pose further uncertainty due to their variable biological behavior and lack of prospective data evaluating PBI; these tumors are generally managed with wide local excision and, in select cases, adjuvant radiotherapy [69]. Finally, ductal carcinoma in situ (DCIS) was included in a only a handful of randomized controlled trials [26,30,50,53,56]. Subgroup analyses from the RAPID and NSABP B39/4013 trials showed minimal differences in IBTR rates between patients treated with PBI and those receiving WBI. Given the low local recurrence risk for small, low to intermediate-grade DCIS, these patients may be suitable candidates for PBI. However, high-grade or larger lesions (>2 cm) require further study before routine PBI use can be recommended [22].

Together, these findings underscore the importance of histological subtype in guiding the selection of the optimal breast irradiation technique. While accelerated PBI is well supported for invasive ductal carcinoma with favorable molecular features, its applicability in other histologies—such as invasive lobular carcinoma or phyllodes tumors—remains poorly defined due to limited representation in clinical trials and inherent challenges in tumor bed delineation. In such cases, whole-breast irradiation, with or without a boost, may offer more comprehensive coverage and remains the preferred approach until further evidence supports the safe and effective use of accelerated PBI in this subgroup [69].

### 3.9. Age

Age is a key determinant of recurrence risk, with younger patients often presenting with more biologically aggressive tumors [22]. However, the impact of age on IBTR after PBI remains inconclusive, primarily due to the underrepresentation of younger patients in randomized trials. Most PBI studies enrolled patients over the age of 50, while fewer than one thousand patients aged 40–49 were included across trials. Subgroup analyses of this 40–49 age group have shown no significant increase in IBTR rates compared to older cohorts [22,65,70], while patients aged >50 showed favourable outcomes. While PBI may be appropriate for carefully selected patients aged 40–49, the NSABP-B39/RTOG 0413 trial reported a non-significant trend favoring WBI in this group [30]. In contrast, patients under the age of 40 were rarely included in PBI trials, and there is currently insufficient evidence to recommend its use in this population. Based on the available data, PBI is strongly recommended for postmenopausal women aged ≥50 years, may be considered for select patients aged 40–49 years, and is not recommended for patients under 40 [22].

## 4. Limitations and Considerations for PBI in Clinical Practice

While RCTs provide strong evidence supporting PBI over WBI for selected early-stage breast cancer patients, several limitations in the current literature warrant consideration. A key challenge in PBI research is the variability in technical factors influencing outcomes, including treatment modality, technique, fractionation regimen, dose per fraction, and total dose. RCTs demonstrate considerable variation in these parameters, which directly impacts toxicity and cosmetic outcomes. Additionally, inconsistencies in toxicity and cosmesis assessments—such as differences in scoring scales, incomplete data collection, and limited patient subsets—complicate efforts to draw broad, generalizable conclusions [22,65]. Another limitation is the lack of direct comparisons between PBI techniques. None of the included RCTs have directly evaluated oncologic or cosmetic outcomes of individual PBI modalities against one another. As a result, the relative effectiveness of different approaches remains unclear as most trials compare distinct PBI techniques against WBI rather than with each other [22,23,65]. The evolution of WBI techniques further complicates interpretation. Many PBI trials were conducted when WBI relied on older regimens, such as 3D-CRT [30,38,49,50,51,53,55] and telecobalt [27], rather than modern treatment machines [50,52], making it challenging to translate past findings into contemporary clinical practice. Clinical precision is a critical consideration in PBI as its success depends on accurate localization of the target volume. The localization of the target volume is further complicated by the increasing use of oncoplastic surgical techniques, which can reposition breast tissue, leading to increased uncertainty of tumor bed delineation. While PBI minimizes normal tissue exposure by confining radiation to a smaller volume, this narrower margin increases the risk of geographic miss, potentially leading to higher local recurrence rates [23]. This concern is particularly relevant in the postoperative setting, where variability in tumor bed contouring among clinicians has been well documented and may impact treatment accuracy. Tumor bed markers, such as gold fiducials or titanium surgical clips, have been used with some success to reduce interobserver variability in tumor bed delineation [42]. For accurate tumor bed localization, recommendations suggest using between 4 and 6 markers to mark the extents of the tumor bed cavity. However, in clinical practice, there is significant variability in both the location and number of markers used [20,42]. The use of hypofractionated and accelerated PBI schedules raises concerns about long-term toxicities, including fibrosis and poor cosmetic outcomes, particularly when fractionation is not carefully optimized [22,23,53]. For instance, the RAPID trial demonstrated that a highly hypofractionated twice-daily regimen led to worsened fibrosis and suboptimal cosmetic outcomes, underscoring the need to balance efficacy with safety in treatment design.

This concern about fractionation and toxicity is further supported by recent long-term data from a dose-escalation trial that evaluated three different twice-daily dose regimens (32 Gy, 36 Gy, and 40 Gy). While all three dose levels achieved acceptable local control with 15-year local failure rates of 6.9%, 5%, and 3.9%, respectively, the higher doses were associated with significantly worse cosmetic outcomes [71]. These findings demonstrate a clear dose–response relationship for toxicity, suggesting that lower total doses may optimize the balance between tumor control and cosmetic outcomes. Additionally, the logistical challenges of twice-daily treatment schedules, which require at least 6 h intervals between fractions, may limit their practical implementation and patient acceptance.

### 4.1. Future Directions in PBI

#### 4.1.1. MRI-Guided Radiotherapy (MRgRT)

While PBI has significantly advanced the standard of care for early-stage breast cancer, several challenges persist—particularly in the postoperative (adjuvant) setting. Conventional EBRT-based PBI requires planning target volume (PTV) margins to account for inter- and intra-fractional anatomical variations [72,73,74]. Although these margins help ensure adequate target coverage, they also increase the volume of irradiated normal tissue, potentially exposing critical structures such as the heart, lungs, and skin [72,75].

Accurate tumor bed localization is essential for effective PBI, yet this remains challenging with conventional imaging. CT, the most commonly used modality for radiotherapy planning, lacks the soft tissue contrast needed to clearly delineate the tumor bed—particularly in the post-lumpectomy setting. Radiopaque surgical clips can aid localization, but their potential for postoperative migration may compromise accuracy. To address this, the ASTRO clinical practice guidelines provide standardized recommendations for target delineation. These guidelines suggest expanding the clinical target volume (CTV) by 1–1.5 cm around the tumor bed, with larger expansions considered in cases of close surgical margins. A 1 cm margin around the CTV is generally recommended for the PTV, although this may be reduced when daily image guidance is used [22]. MRI addresses many of the limitations associated with CT. Compared to CT, MRI offers superior soft tissue contrast, allowing for more accurate tumor bed visualization. This enhanced imaging quality reduces interobserver variability and improves CTV definition, even in the absence of surgical clips. MRgRT is a relatively new approach that combines high soft tissue contrast and real-time imaging with advanced radiation delivery techniques [4,76,77]. This integration enables precise tumor bed visualization and supports daily treatment adaptation. Unlike static treatment plans that rely on pre-treatment imaging, MRgRT facilitates online adaptive radiation therapy (ART) in which treatment plans are reoptimized in real time while the patient remains on the treatment table. This allows for the use of tighter PTV margins [75,78], which is particularly beneficial in PBI, where target volumes may decrease over time due to seroma resorption. In breast cancer, MRgRT holds significant promise for enhancing the precision of PBI by improving target coverage while reducing the irradiation of surrounding healthy tissue [72]. However, further research is needed to validate its long-term impact, particularly in terms of local control, toxicity reduction, and cost-effectiveness.

#### 4.1.2. Neoadjuvant PBI

Neoadjuvant (preoperative) PBI is emerging as a promising alternative to the conventional postoperative approach for early-stage breast cancer [79]. While BCS followed by postoperative radiotherapy remains the standard of care, challenges such as surgical delays, treatment-related toxicities, and variability in target delineation highlight the need for improved strategies. By targeting the gross tumor volume (GTV), as seen on imaging before surgery, neoadjuvant PBI allows for the precise delineation of high-risk tissue while the tumor remains in situ. This approach improves target definition, reduces clinical target volumes, can enhance dose conformity, and reduces radiation exposure to surrounding healthy tissue [80]. Additionally, removing the irradiated tumor at the time of surgery mitigates the risk of fibrosis and necrosis, leading to better cosmetic outcomes [79,81,82,83,84]. Early-phase trials have demonstrated its feasibility, safety, and efficacy, with some studies reporting 100% good to excellent cosmesis and minimal toxicity, even when using large PTV margins [79,82]. For instance, the phase II PAPBI trial, which delivered 40 Gy in 10 daily fractions with a 2.5 cm PTV margin, achieved excellent local control (only two recurrences) and 100% good to excellent cosmesis at 3 years [82].

Compared to postoperative PBI, neoadjuvant PBI also offers greater treatment consistency by avoiding the uncertainties associated with lumpectomy cavity changes and breast distortion after surgery [78,85]. With the tumor still in situ, neoadjuvant PBI reduces interobserver variability in target volume delineation, resulting in smaller treatment margins, improved dose conformity, and decreased irradiation of healthy tissue [83,86]. Additionally, because a smaller volume of tissue is treated, neoadjuvant PBI may allow for higher dose delivery per fraction, making ultra-hypofractionated RT a feasible approach that could reduce treatment burden and, in turn, improve patient adherence to RT treatment [4]. However, despite its potential, key challenges remain. Current data are largely derived from phase I/II trials, as summarized in Table 3, with limited follow-up, leaving uncertainties regarding long-term oncologic safety [81,83,84,87,88,89,90,91]. Additionally, the variability in dose fractionation schedules—ranging from 40 Gy in ten fractions to single-dose protocols (e.g., 21 Gy in one fraction)—complicates cross-study comparisons and hinders the development of unified clinical guidelines. Future research must address these gaps through large-scale RCTs with extended follow-up to determine the optimal fractionation strategy and long-term efficacy of neoadjuvant PBI.

## 5. Conclusions

PBI represents a significant advancement in the management of early-stage breast cancer, offering a highly targeted approach that reduces treatment-related toxicity while maintaining oncological safety. Multiple RCTs using various PBI techniques have demonstrated oncologic equivalence to the current gold standard treatment, WBI, underscoring the potential for PBI to become an integral component of clinical practice. However, a robust evaluation of the comparative toxicity between PBI and WBI remains challenging due to variability in dose fractionation regimens and delivery methods and parameters across studies. As radiation techniques and fractionation schedules continue to evolve, further research is essential to establish standardized protocols and minimize treatment-related risks. A cross-comparison of PBI modalities—including EBRT, brachytherapy, and intraoperative radiotherapy—is also critical to identify the most effective and patient-centered approaches for early-stage breast cancer treatment. Simultaneously, treatment strategies are shifting toward de-escalating local therapy, both surgically and with radiotherapy. Optimizing patient selection criteria and refining treatment techniques will play a key role in this evolution. Neoadjuvant PBI offers a unique opportunity to enhance patient outcomes by targeting the intact tumor while minimizing treatment-related complications. Furthermore, the advent of MRI-guided radiotherapy, with its superior precision and real-time adaptive planning capabilities, offers a promising avenue for future innovation. Future research exploring the integration of MRI-guided radiotherapy into PBI protocols is warranted to maximize its clinical utility and further advance patient-centered care.

## Figures and Tables

**Table 1:** RCTs comparing PBI and WBI.

Trial Name/Study Name	Inclusion Criteria	Study Design	Number of Patients	Technique Used	Dose	Follow-Up Time	IBTR Rate, WBI vs. PBI %)	Toxicity	Cosmetic Outcome	Overall Survival (OS), WBI vs. PBI
Budapest [27]	≥40 yrs, stage I-II, ≤20 mm, negative margins, cN0/pN0/pN1mi, grade 1–2	RCT	258	HDR brachy or electron beam vs. WBI	PBI: 36.4 Gy/7 fx (HDR), 50 Gy/25 fx (electron) WBI: 50 Gy/25 fx	Median 10.8 yrs (1.5–13.5)	5.9% (WBI) vs. 5.1% (PBI)	No difference in late toxicity	Better in PBI group	No difference * (79.7% vs. 82.1%)
GEC-ESTRO [56]	≥40 yrs, stage 0–IIA, ≤30 mm, negative margins, cN0/pN0/pNmi	RCT	1184	HDR/PDR Brachy vs. WBI	PBI: HDR 32 Gy/8 fx, PDR 50 Gy/continuous; WBI: 50 Gy/25–28 fx + 10 Gy boost	Median 6.6 yrs (5.8–7.6)	0.92% (WBI) vs. 1.44% (*p* = 0.42) (PBI)	Lower late skin toxicity (PBI)	Better in PBI group	No difference * (95.6% vs. 97.3%)
GEC-ESTRO [26]						Median 10.4 yrs (IQR 9.1–11.3)	1.58% (WBI) vs. 3.51% (PBI)	Lower late skin toxicity (PBI)	Better in PBI group	No difference * (89.5% vs. 90.5%)
TARGIT [35]	≥45 yrs, operable invasive BC (T1–small T2, N0–1, M0), suitable for BCS	RCT	3451	IORT (intrabeam) vs. WBI	PBI: 20 Gy at tumor bed surface; WBI: 50 Gy/25 fx	Median 2.5 yrs (IQR 12–52 mo)	1.3% (PBI) vs. 3.3% (*p* = 0.042) (WBI)	Lower acute skin toxicity (PBI) and higher long-term fat necrosis and scar calcification (PBI)	Worse in PBI group	No difference * (3.9% vs. 5.3%)
ELIOT [36]	48–75 yrs, early BC, ≤2.5 cm, BCS candidates	RCT	1305	IORT vs. WBI	PBI: 21 Gy single dose; WBI: 50 Gy/25 fx	Median 5.8 yrs (4.1–7.7)	4.4% (PBI) vs. 0.4% (*p* = 0.0001) (WBI)	Lower skin toxicity (PBI)	No difference	No difference * (96.8% vs. 96.9%)
Barcelona [49]	≥60 yrs, invasive ductal carcinoma, ≤30 mm, cN0/pN0	RCT	102	3D-CRT PBI vs. WBI	PBI: 37.5 Gy/10 fx BID; WBI: 48 Gy/24 fx + 10 Gy boost	Median 5 yrs (IQR: NR)	0% (PBI) vs. 0% (WBI)	Lower acute toxicity (PBI)	66.7% WBI vs. 56.5% PBI rated excellent	No difference *
Florence [55]	≥40 yrs, early BC, ≤2.5 cm, BCS candidates	RCT	520	IMRT PBI vs. WBI	PBI: 30 Gy/5 fx; WBI: 50 Gy/25 fx + 10 Gy boost	5.0 yrs (3.4–7.0)	1.5% (PBI and WBI group)	Lower acute/late toxicity (PBI)	Better in PBI group (*p* = 0.045)	No difference * (HR 0.99; 95% CI 0.83–1.18)
Florence [50]					PBI: 30 Gy/5 fx; WBI: 50 Gy/25 fx + 10 Gy boost	Median 10.7 yrs (1.4–14.8)	2.5% (WBI) vs. 3.7% (PBI) (*p* = 0.40)	Lower acute/late toxicity (PBI)	Better in PBI group	No difference * (91.9% both)
DBCG-APBI [51]	Women ≥60 years with ER-positive, HER2-negative, grade 1–2, nonlobular breast cancer (<21 mm, pT1pN0M0), ≥2 mm margins	RCT	865	NR	WBI and PBI: 40 Gy/15 fx	Median 5.0 years (4.1–5.1)	9.7 (WBI) vs. 5.1% (PBI)	NR	Better in PBI group 89%PBI v 85% WBI	No difference * (86.5% vs. 89.3%)
UK-IMPORT LOW [52]	Women ≥50 years with unifocal invasive ductal (grade 1–3, pT1–2, ≤3 cm, pN0–1), ≥2 mm margins	RCT	2018	IMRT	WBI and PBI: 40 Gy/15 fx	Median 6.0 years (5.1–6.9)	1.1% (WBI) vs. 0.5% (PBI)	Lower acute/late toxicity (PBI)	NR	No difference *
RAPID [53]	≥40 yrs, DCIS/invasive BC, BCS with clear margins, node-negative	RCT	2135	3D-CRT/IMRT PBI vs. WBI	PBI: 38.5 Gy/10 fx BID; WBI: 42.5 Gy/16 fx or 50 Gy/25 fx	Median 8.6 yrs (7.3–9.9)	2.6% (PBI) vs. 3.5% (WBI)	Lower acute toxicity, higher late toxicity (PBI)	Worse in PBI group	No difference *
IRMA [54]	≤3 cm, negative nodes or 1–3 nodes, CTV <30% breast volume	RCT	3309	3D-CRT/IMRT PBI vs. WBI	PBI: 38.5 Gy/10 fx BID; WBI: 50 Gy/25 fx or 40 Gy/15 fx	Median 5.6 yrs (4.0–8.4)	NR	Higher late toxicity (PBI)	Worse in PBI group	No difference * (97.2% vs. 97.4%)
NSABP-B39/RTOG 0413 [30]	≥18 yrs, early-stage BC, ≤3 cm, node-negative or ≤3 nodes	RCT	4216	3D-CRT/Brachy PBI vs. WBI	PBI: 34 Gy (Brachy) or 38.5 Gy (EBRT); WBI: 50 Gy/25 fx	Median 10.2 yrs (7.5–11.5)	3.9% (WBI) vs. 4.6% (PBI)	Similar moderate/high toxicity	No difference	No difference * (97.1% vs. 96.7%)

Abbreviations: IBRT = ipsilateral breast tumor recurrence; IMBT = interstitial multicatheter brachytherapy; IMRT = intensity modulated radiotherapy; NR = not reported; PBI = partial breast irradiation; 3D-CRT = 3D conformal radiotherapy; WBI = whole-breast radiotherapy, OS = overall survival; IORT = intraoperative radiation therapy; IQR = interquartile range; BC = breast cancer; BCS = breast conservation surgery; RCT = randomized controlled trial; HDR Brachy = high-dose-rate brachytherapy; PDR Brachy = pulsed-dose-rate brachytherapy; BID = twice a day; CI = confidence interval. * = No statistical differences reported.

**Table 2:** Guidelines for patient selection for PBI.

Criteria	GEC-ESTRO [65]	ASTRO [22]	ABS [41]
Age	≥50 years	≥50 years	≥45 years
Lymph Node Status	Negative	Negative	Negative
Lymphovascular Invasion	None	None	None
Tumor Size	≤3 cm	≤2 cm grade 1–2	≤3 cm
Margins	≥2 mm	≥3 mm	Not specified
Histology	Invasive ductal, ER+/− (excluding invasive lobular carcinoma or DCIS)	Invasive ductal, ER+, DCIS (invasive lobular carcinoma excluded)	All invasive and DCIS (ER status irrelevant)
Multifocal or Unifocal Disease	Unifocal	Unifocal	Unifocal
Neoadjuvant Therapy	No neoadjuvant chemotherapy	No Neoadjuvant Therapy	Not specified

Abbreviations: ABS = American Brachytherapy Society, ASTRO = American Society of Radiation Oncology, GEC-ESTRO = Groupe Européen de Curiethérapie/European Society for Therapeutic Radiology and Oncology.

**Table 3:** Summary of clinical trials evaluating treatment approaches when using neoadjuvant PBI only.

Study	Description/Endpoint	Equipment Used	Treatment
Preoperative Accelerated Partial Breast Irradiation for Early-Stage Breast Cancer [83]	Evaluate feasibility of utilizing 3D CRT PBI in preoperative setting followed by BCS	3D CRT	38.5 Gy/10 fx
Preoperative robotic radiosurgery for early breast cancer: Results of the phase II ROCK trial [88]	Evaluate safety and feasibility of single-fraction preoperative RT in early-stage breast cancer and identify biological and clinical predictors of treatment outcomes, including toxicity, pathological response, and imaging biomarkers	CyberKnife^®^ RT	21 Gy/1 fx
Tumor response 3 months after neoadjuvant single-fraction radiotherapy for low-risk breast cancer [87]	Evaluate feasibility of delivering single-fraction RT and safety	NR	21 Gy/1 fx
Evaluation of Early Response to Preoperative Accelerated Partial Breast Irradiation (PAPBI) trial [81]	Evaluate biomarkers for early response to RT in breast cancer	3D CRT, IMRT or VMAT	40 Gy/10 fx
Single-dose radiation, then lumpectomy, for early breast cancer: the SIGNAL trial [90]	Evaluate feasibility, toxicity, surgical, oncologic, and cosmetic outcomes of single-fraction preoperative PBI	Cone-Beam CT	21 Gy/1 fx
Single Pre-Operative RT (SPORT) for low-risk BC [92]	Evaluate toxicity, surgical, oncologic, and cosmetic outcomes of single-fraction preoperative PBI	VMAT	21 Gy/1 fx
Single Dose Ablative RT for Early-Stage BC (ABLATIVE I) [84]	Evaluate feasibility, safety, and efficacy of preoperative RT in single fraction for breast cancer patients and collect data on response monitoring	MRgRT	20 Gy/1 fx tumor with 15 Gy to tumor bed
Preoperative Single-Fraction Radiotherapy in Early-Stage Breast Cancer [93]	Evaluate physician-reported rates of cosmesis	CyberKnife^®^ RT	18–24 Gy/1 fx to GTV
Magnetic resonance imaging-guided single-fraction preoperative radiotherapy for early-stage breast cancer (the RICE trial): feasibility study [4]	Evaluate feasibility of treatment using single fraction and collect data on response monitoring	MRgRT	21 Gy/1 fx
Prediction of pathologic complete response after single-dose MR-guided partial breast irradiation in low-risk breast cancer patients: the ABLATIVE-2 trial [3]	Evaluate response monitoring after 12-month single-fraction treatment	MRgRT	20 Gy/1 fx tumor with 15 Gy to tumor bed
SABER study for selected early-stage BC [94]	Evaluate safety, feasibility, and dose-limiting toxicity of up to four dose levels of stereotactic ablative breast radiotherapy, with surgery planned 4–6 weeks post-treatment, assessing toxicity, pathologic response, and treatment feasibility	NR	Dose Level I: 35 Gy (5 fx of 7 Gy) Dose Level II: 40 Gy (5 fx of 8 Gy) Dose Level III: 45 Gy (5 fx of 9 Gy) Dose Level IV: 50 Gy (5 fx of 10 Gy)

Abbreviations: NR = not reported; PBI = partial breast irradiation; 3D-CRT = 3D conformal radiotherapy; WBI = whole-breast radiotherapy; Gy = gray; fx = fraction; MRgRT = MRI-guided radiotherapy; RT = radiation therapy; BC = breast cancer; BCS = breast conservation surgery; CT = computed tomography; VMAT = volumetric modulated arc therapy; IMRT = intensity modulated radiotherapy.

## Abbreviations

The following abbreviations are used in this manuscript:

BCT	Breast-Conserving Therapy
BCS	Breast-Conserving Surgery
WBI	Whole-Breast Irradiation
PBI	Partial Breast Irradiation
IORT	Intraoperative Radiotherapy
EBRT	External Beam Radiotherapy
3D-CRT	Three-Dimensional Conformal Radiotherapy
IMBT	Intensity-Modulated Brachytherapy
LINAC	Linear Accelerator
MRgRT	Magnetic Resonance-Guided Radiotherapy
GTV	Gross Tumor Volume
PTV	Planning Target Volume
Gy	Gray (Unit of Radiation Dose)
RCT	Randomized Controlled Trial
IBTR	Ipsilateral Breast Tumor Recurrence
ABS	American Brachytherapy Society
ASTRO	American Society for Radiation Oncology
GEC-ESTRO	Groupe Européen de Curiethérapie—European Society for Radiotherapy and Oncology
MRI	Magnetic Resonance Imaging
ER	Estrogen Receptor
